# Distinct bone metabolic networks identified in *Phospho*1^−/−^ mice vs. wild type mice using [^18^F]FDG total-body PET

**DOI:** 10.3389/fmed.2025.1597844

**Published:** 2025-05-21

**Authors:** Abigail F. Hellman, Paul S. Clegg, Colin Farquharson, José Luis Millán, Carlos J. Alcaide-Corral, Karla J. Suchacki, Adriana A. S. Tavares

**Affiliations:** ^1^School of Physics and Astronomy, University of Edinburgh, Edinburgh, United Kingdom; ^2^The Roslin Institute, University of Edinburgh, Midlothian, United Kingdom; ^3^Sanford Burnham Prebus, Medical Discovery Institute, La Jolla, CA, United States; ^4^University/British Heart Foundation (BHF) Centre for Cardiovascular Science, The Queen's Medical Research Institute, University of Edinburgh, Edinburgh, United Kingdom; ^5^Edinburgh Imaging, University of Edinburgh, Edinburgh, United Kingdom; ^6^Scotland's Rural College, The Roslin Institute, Midlothian, United Kingdom

**Keywords:** positron emission tomography, network analysis, systems biology, bone, PHOSPHO1

## Abstract

**Introduction:**

Total-body PET is a recent development in clinical imaging that produces large datasets involving multiple tissues, enabling the use of new analytical methods for multi-organ assessments, such as network analysis—a well-developed method in neuroimaging. The skeletal system provides a good model for applying network analysis to total-body PET, as bone serves many classical whole-body functions as well as being an endocrine regulator of metabolism. Previous reports have suggested an association between the expression of bone-specific phosphatase, orphan 1 and disorders of altered energy metabolism such as obesity and diabetes. Here, we explore how lacking phosphatase, orphan 1 affects the skeletal metabolic networks of mice as a test approach for deploying network analysis in total-body PET.

**Methods:**

We retrospectively analysed [^18^F]fluorodeoxyglucose total-body PET/CT images from six 13-week-old wild type mice, three 22-week-old wild type mice, and three 22-week-old *Phospho1*^−/−^ mice. Pearson correlation networks were created using the dynamic data from seven bone regions, with a Pearson threshold of *r*>0.6 (significant at *p* < 0.005).

**Results:**

The bone metabolic networks of 13-week-old wild type mice were found to robustly resist changes to the data from different PET measurements, increased noise, and shortened scan length. Key features were repeatedly observed, namely that all bones except the spine are highly inter-correlated, while the spine has minimal correlation to other bones. When network analysis was used to compare the three cohorts, the older wild type network had similar features to the young mouse, whereas the *Phospho1*^−/−^ network had increased correlations across all bones. An all-cohort network separated the data into one part including only bones from the wild type mice (13 nodes) and one part only bones from the *Phospho1*^−/−^ mice (8 nodes, 95% separation purity). Within the wild type section, the same bone from each young and old mouse were correlated.

**Discussion:**

We demonstrated network analysis is a promising method for studying whole-body PET, sensitive to dynamic details in the data without relying on assumptions or modelling. The proposed method could be applied to other total-body PET data—of healthy and diseased subjects, with different radiotracers, and more—to further elucidate tissue interactions at a systems level.

## 1 Introduction

Total-body positron emission tomography (TBPET) scanners have a larger axial field-of-view compared to traditional PET, producing large datasets involving multiple tissues. TBPET has seen frequent use in pre-clinical research, but it is relatively new in clinical imaging (1–3). As a result of scanning the entire body at once, TBPET provides an opportunity to study systems biology and systemic disease. Many diseases are well-known for being systemic, such as vascular disease or metastasised cancer, and TBPET could help in better understanding these conditions. Furthermore, some diseases that have long been thought of as localised to one organ are being discovered to have a systemic effect (1). For example, recent evidence has shown that there is a significant link between neurological and cardiovascular health known as the heart-brain axis (4). Cardiovascular diseases are associated with higher risk of brain diseases while brain diseases are often linked with failure of the autonomic nervous system (4). There has even been research into treatment links across organ systems. One example is the gut-brain axis where cognitive behavioural therapy has been observed to positively affect gastrointestinal disorders such as irritable bowel syndrome (1). It is systemic links such as these where TBPET can aid in understanding how different organs and organ systems interact in healthy and disease states.

The traditional methods for PET image analysis, such as calculating standardised uptake value (SUV) or performing pharmacokinetic analysis, each hold an important place in PET work and are applicable to TBPET. However, they each come with their own trade-offs. SUV is a semi-quantitative method of assessing the normalised radiotracer uptake in a given region of interest, and is particularly useful in clinical work for lesion detection and the evaluation of therapeutic efficacy (5). However, there are many factors, such as noise and patient motion, that can affect the accuracy of SUV (6, 7). In addition, SUV does not provide information on the underlying physiological changes that lead to unusual tracer uptake; for example, SUV values may be high due to increased blood flow through an organ and not due to increased uptake by an organ (8). On the other hand, pharmacokinetic analysis is the gold standard for PET, as a quantitative method that uncovers information about the delivery, uptake, retention, and clearance of radiotracer by tissue (7). It is extremely useful for determining the physiological origin of significant changes in the PET scan, but requires blood sampling throughout a longer dynamic scan, where a subject is imaged at multiple time points after injection (8). This makes it an invasive and time-consuming process, and thus not ideal for clinical use. While SUV is quick but semi-quantitative, pharmacokinetics is very informative but laborious. Contrastingly, network analysis is a fully quantitative method of image analysis that can be easily implemented and may prove particularly useful for studying systems biology with TBPET.

Network analysis is a method of correlation analysis that produces representative networks, or graphs, of quantified similarities and differences in image data from different tissues and subjects. Network analysis is not yet a common analysis method for multi-organ studies. However, it has seen much use in single-organ research, particularly of the brain (9, 10). Networks have been used to explore both the structure and functionality of the brain using imaging methods such as magnetic resonance imaging (MRI) and PET which both can provide information as to how signals from different parts of the brain are correlated (11–13). Inferences can then be made about the relationships and interactions that occur between brain regions. Interestingly, functional and structural networks of patients with brain diseases such as schizophrenia or Alzheimer's have also been shown to differ from their healthy counterparts (9). This shows the potential for network analysis to be a useful tool not only for studying pathology, but also as a method of disease diagnosis and prognosis tracking. The goal of applying network analysis to TBPET is similar to that of brain studies—to further explore correlations in physiological behaviours between different areas of the body and how these correlations may change with disease.

Network analysis with clinical total-body and whole-body PET has shown initial promising results. It has been shown with group-level analysis of static TBPET data that metabolic networks of lung cancer patients are significantly different from healthy subjects (14, 15). Lung cancer patient networks are characterised by a decrease in efficiency, which implies that lung cancer disrupts metabolic regulation and coordination between organs (14). There has also been exploratory work on using network analysis to understand, diagnose, and manage cachexia with static whole-body PET data (16). Other studies have used dynamic whole-body PET to explore methods for performing individual-level analysis with healthy subjects (17), and for comparing diabetic and non-diabetic patients (18). These analyses highlight the increasingly relevant field of systemic connectivity in PET research, largely driven by the development of extended field of view imaging (19).

Here we present a method for applying network analysis to dynamic TBPET data, using the radiotracer [^18^F]fluorodeoxyglucose ([^18^F]FDG) for assessing glucose metabolism in mice. Data from different bones were compared, because the skeleton serves as a good model for studying complex interactions throughout the body. The skeleton performs many well-known functions throughout the entire body such as organ protection, calcium and phosphorous storage, and locomotion. Additionally, bones also have recently been discovered to serve important endocrine functions (20). For example, the bone mineralization phosphatase, Phosphatase, Orphan 1 (PHOSPHO1) has been implicated to play a role in metabolic regulation in both mice and humans (21–26). *Phospho1*^−/−^ mice, which lack PHOSPHO1, have been shown to have improved glucose homeostasis compared to their wild type (WT) littermates, and resist high-fat-diet-induced weight gain and diabetes (21, 27, 28). Here we not only seek to test the robustness of our proposed method of analysing TBPET data with network analysis, but also to apply the method for comparing *Phospho1*^−/−^ mice and WT mice of varying ages.

## 2 Materials and methods

### 2.1 Animals

The data analysed here comes from two prior pre-clinical studies, one of which was previously presented in the context of skeletal metabolism networks (29). This first cohort is composed of six adult male mice of the strain C57BL/6JCrl (13.5 ± 0.2 weeks, 29.4 ± 1.6 g, mean±SD) which were housed at 22 − 23°*C* on a 12 h light/dark cycle. The mice had free access to food and water, until they were fasted for 4 h prior to PET/CT acquisition (29). These mice will be referred to here as young wild type (WT). The second cohort involved six adult male mice, older than the first cohort (22.4 ± 0.5 weeks, 31.6 ± 1.9 g, mean ± SD), comprised of three WT mice and three *Phospho1* null mice (27). These mice were a hybrid strain of C3HeB/FeJ (providing the mutation) bred to C57BL/6 (30). Both cohorts were maintained under the same living conditions, and similarly fasted for 4 h prior to imaging (27). These animals will be referred to here as the old WT mice and the *Phospho1*^−/−^ mice—or the old mice cohort all together.

All animal experiments were approved by the University of Edinburgh's named veterinary surgeon and named animal care and welfare officer (NACWO), with animals maintained in accordance with the UK Home Office code of practise.

### 2.2 Image acquisition

**Figure 1:**
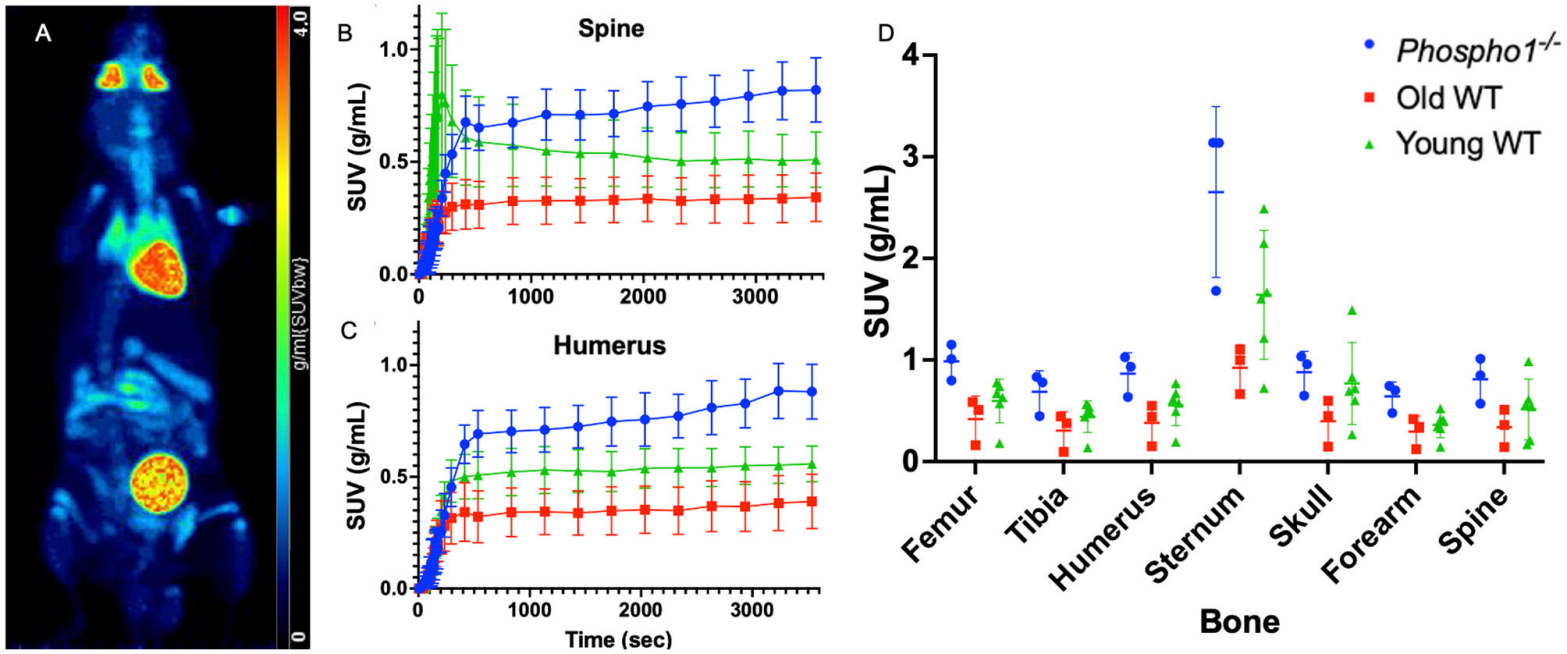
Traditional static SUV analysis yields no statistically significant differences between cohorts. **(A)** A maximum intensity projection of total-body PET data (120 min) for a 13-week-old wild type mouse. **(B)** Group SUV curves of the whole spine region for each of the trial groups: *Phospho1*^−/−^ (blue), old WT (red), and young WT (green). **(C)** Group SUV curves of the whole humerus region for each of the three trial groups. **(D)** Comparison of the mean SUV at equilibrium across the three trial groups with two-way ANOVA returned no statistically significant differences in any of the seven bones. Data are presented as mean ± SEM, *n* = 3 for *Phospho1*^−/−^, *n* = 3 for old WT, and *n* = 6 for young WT.

Mice were weighed and anaesthetised with a pre-set mixture of 0.5/0.5 L/min of oxygen/nitrous oxide and 2 or 2.5% isoflurane (2% for the young mice, 2.5% for the old mice). They were then transferred to the microPET/CT scanner (nanoPET/CT, Mediso, Hungary). General anaesthesia was maintained throughout the entire PET/CT acquisition, and temperature and respiration rate were monitored. All 12 mice received a tail vein intravenous bolus injection of [^18^F]fluorodeoxyglucose ([^18^F]FDG, young: 15.1 ± 5.9 MBq, old: 8.1 ± 3.8 MBq, mean±SD). The radiotracer was produced using standard radiolabelling methods (31). After radiotracer administration, the young WT mice underwent a 120-min total-body PET scan (Figure 1A) and the old WT mice and *Phospho1*^−/−^ mice underwent a 60-min scan. The young WT mice received a longer scan than the old WT mice because they are from two different previous studies. The study protocol the young WT data is from was designed to compare dosimetry of tracers over an extended scan period. The PET scans were followed by a CT scan (semi-circular full trajectory, maximum field of view, 360 projections, 50 kVp, 300 ms and 1:4 binning). The CT data were used to perform attenuation correction on the PET images. For the young WT mice, the PET images were reconstructed into 6 × 30, 3 × 60, 2 × 120, 10 × 300, and 6 × 600 s frames using Mediso's iterative Tera-Tomo 3D reconstruction algorithm, with the following settings: four iterations, six subsets, full detector model, low regularisation, spike filter on, voxel size 0.4 mm, and 400–600 keV energy window (29). For the old WT mice, the PET images were reconstructed into 18 × 10, 2 × 30, 1 × 60, 2 × 120, 10 × 300 s and the Mediso Tera-Tomo 3D reconstruction algorithm was employed with the same settings. PET data were corrected for random coincidences, scatter, and attenuation.

### 2.3 Image processing

The 12 PET/CT scans were processed using the image analysis software PMOD version 4.4 (PMOD Technologies, Switzerland) (32). Firstly, the PET data was corrected for the time delay between the radiotracer injection and the scan start time. Then, the PET images were re-sliced to match the CT images, bringing them into a common coordinate space for precise segmentation. For each mouse, seven bones were segmented on the CT scan: the skull, spine, sternum, humeri, forearms (radius and ulna), femurs, and tibiae. Two segmentation methods were used. The first method was only performed on the six young WT mice as a way to validate the networks under a low signal-to-noise ratio condition. In this method of segmentation, two to three cuboid volumes of interest (VOIs), of one cubic millimetre in size, were placed in each bone. Each cube contained only bone as verified by a Hounsfield unit (HU) threshold of 332 (29, 33). The VOIs were then transferred onto the PET scan, where each cube was made up of 27 pixels in total. Time-activity curves (TACs) were extracted both in terms of measured activity (kBq/ml) and SUV, which was normalised for injected dose and body weight. The TACs were averaged across all pixels and for all cubes within a given bone. This produced seven TACs per mouse for each measurement unit.

In the second segmentation method, applied to all 12 mice, volumes of interest (VOI) were drawn around each of the seven bones and then segmented using HU from the CT to distinguish bone (332–50,000) from bone marrow and other tissues. The VOIs were then transferred onto the PET scan. The data was extracted from the scan in terms of the average SUV for each VOI, again normalised for injected dose and body weight. An average was taken of left and right appendicular bones, giving seven total TACs per mouse for this segmentation method.

### 2.4 Statistical SUV analysis

The average SUV value was calculated from PET frames between 45 and 60 min post-radiotracer injection for all 12 mice. These data are presented as mean with standard deviation for each of the three cohorts. Two-way analysis of variance (ANOVA) with multiple comparisons was performed between cohorts for each bone using Prism version 10.3.0 (GraphPad v10, USA).

### 2.5 Network analysis

Network analysis was performed using the Pearson correlation coefficient (PCC), a linear correlation measure, in Graphia (34). To do this, it is necessary for the PET data to be in tabular format in a comma separated values (CSV) file, where rows are different VOIs and columns are different time points. The Pearson value, *r*, between each TAC is then calculated in Graphia. A graph is then visualised where nodes represent each bone TAC and edges between them represent significant correlations (*r*>0.6, *p* < 0.005) (35, 36). The edges are weighted to the Pearson value. In addition to having a Pearson threshold for edges in each network, a k-nearest neighbours edge reduction algorithm was applied (*k* = 3). This algorithm reduces the total number of edges in the network, retaining only the *k* strongest weighted edges for each node unless there are more than *k* of equally strong weight (34, 37).

To explore the robustness of the network analysis paradigm, many different networks were created through this method. Firstly, one network per mouse for each segmentation method applied to the six young WT mice was created: cuboid VOI kBq/mL data, cuboid VOI SUV data, and whole-bone VOI SUV data. This was done to test network consistency across multiple PET metrics (kBq/mL and SUV) and to evaluate the effect of noise on the networks as the cuboid VOI data has more noise due to the small region size. Additionally, further networks were created with the whole-bone VOI SUV data cropped to 60 min, to assess if reducing the amount of data in this way affected network stability.

In order to compare with the young WT mice, one network for each of the six older mice was created with the whole-bone VOI SUV data. Next, whole-bone SUV data was averaged at each time point within each of the three cohorts to create one average network for each. Finally, the average data was compiled into one network including all the mice. Given the higher number of nodes, it is possible to define smaller communities within this larger network. Communities are defined as node groupings where a given node within a group has a higher probability of being connected to other nodes in its community than any node outside of it (38). Practically, this looks like networks with many connexions between nodes inside a community, and reduced connexions across communities.

## 3 Results

### 3.1 No significant differences identified by statistical SUV analysis

Observational comparison of the mean SUV curves of the three cohorts (Figures 1B, C, Supplementary Figure 1) shows that the *Phospho1*^−/−^ mice are characterised by an overall high radiotracer uptake, while the old WT generally have the lowest uptake of the three groups. Despite any observational differences, when the mean SUV at equilibrium is compared across cohorts for each bone using two-way ANOVA as described in Section 2.4, there is no significant difference (Figure 1D, *p*>0.05).

### 3.2 Network analysis is robust to PET data changes from reduced datasets, different activity measurements, and VOI selection

**Figure 2:**
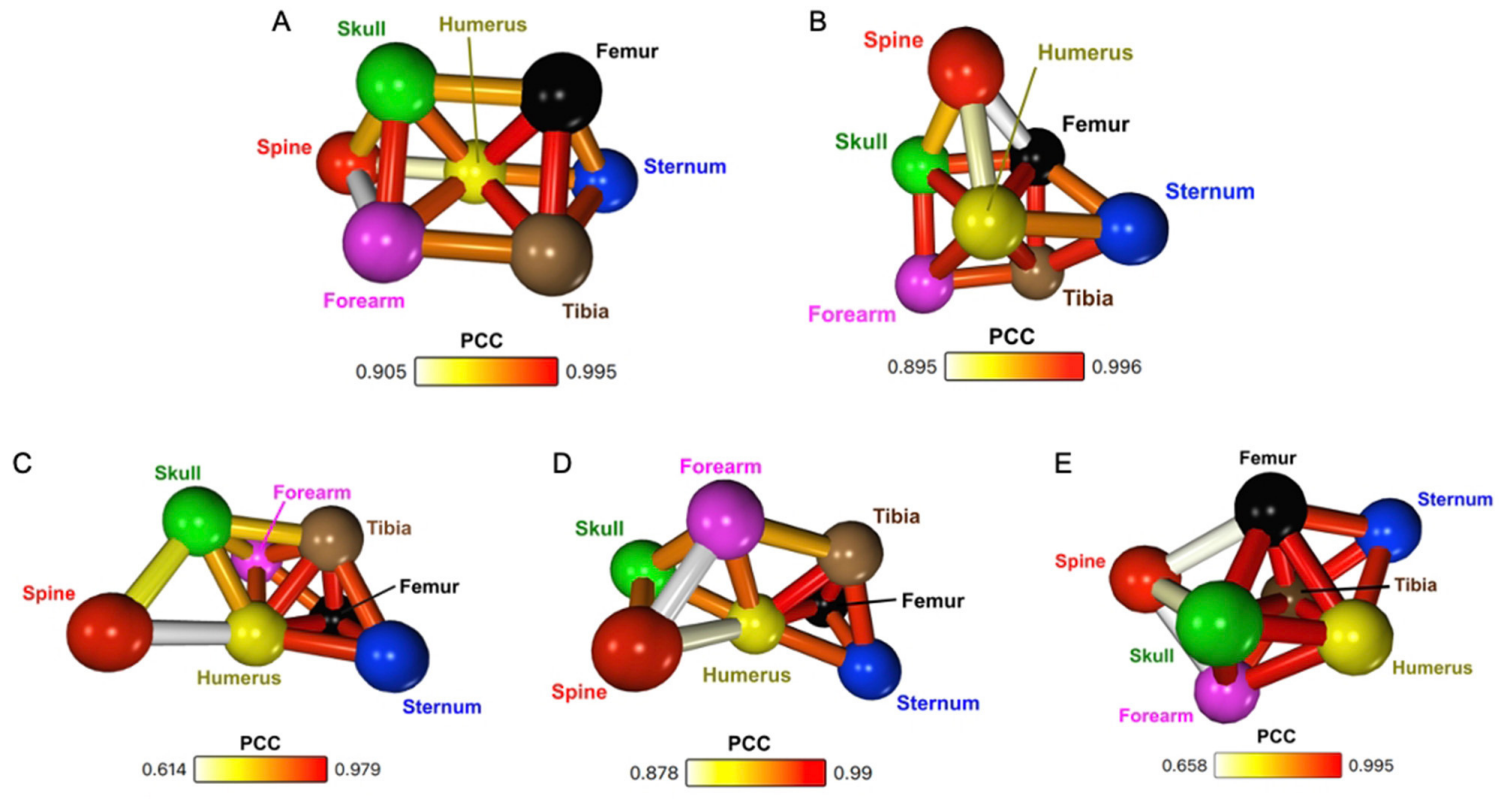
**(A)** Whole-bone dynamic SUV network for a single mouse, including all 120 min of scan time. **(B)** Whole-bone dynamic SUV network for a single mouse, including only data from the first 60 min of scan time. **(C)** Cuboid region dynamic kBq/mL network for a single mouse (60 min). **(D)** Cuboid region averaged dynamic kBq/mL network of six 13-week WT mice (60 min). **(E)** Whole-bone averaged dynamic SUV network of six 13-week WT mice (60 min).

As described in Sections 2.3 and 2.5, network analysis was performed on PET data extracted from the six young WT mice in five different ways. At the individual mouse level, for all six mice, networks were created using different radiotracer uptake measurements (kBq/mL and SUV), different VOI sizes (whole-bone or cuboid), and different timescales (1 and 2 h). An average network of all six mice using whole-bone dynamic SUV for a period of 60 min was also created. The resulting networks, shown in Figures 2A–E, display correlations of *r*>0.6, significant at *p* < 0.001 for the full 2 h of data and *p* < 0.005 for the networks restricted to only 1 h of data. Figures 2A–D are individual mouse networks all from the same single mouse, whereas Figure 2E represents the average of all six young WT mice. The edges in the networks are colour-coded to the weight of the PCC between each node, with the scale given for each. The scale often starts above the minimum Pearson threshold of *r*>0.6 due to the strong nature of the correlations between regions, coupled with the pruning effect of the *k-NN* edge reduction algorithm.

Comparing each of these five networks, a few shared characteristics are apparent. Namely, the long bones (humerus, forearm, femur, tibia) are highly intercorrelated, with high PCC and increased edge density between them. The humerus in particular is a central node in each of the networks, with a high density of edges. Conversely, the spine is often dissimilar from the rest of the network, still connected to other regions, but with low degree and the lowest PCC values in the network. These findings are not only consistent between the young WT networks, but also agree with previously published results (29).

### 3.3 Network analysis identifies and separates different *Phospho1^−/−^* and WT pathologies based on bone glucose metabolism

**Figure 3:**
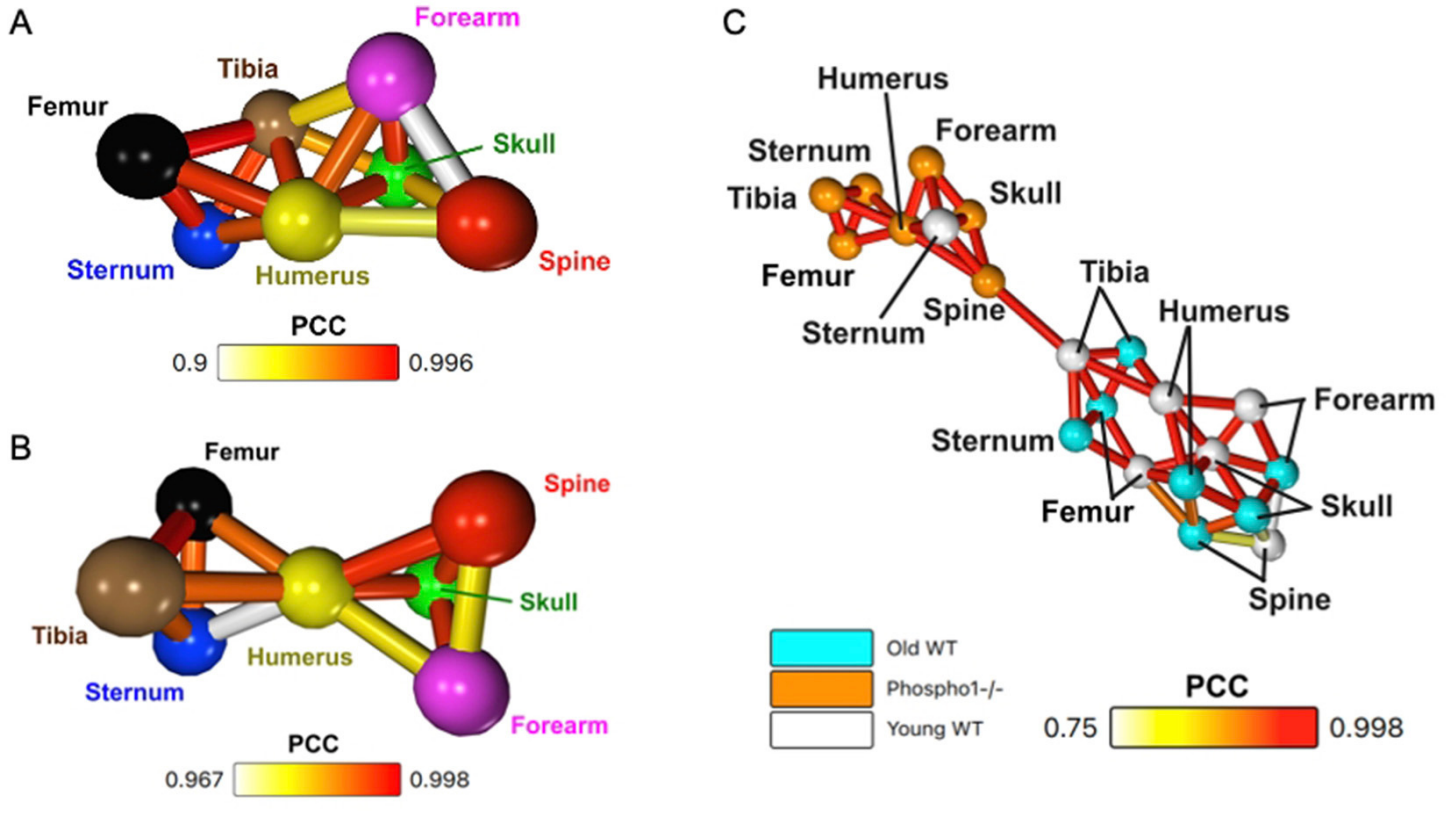
Network analysis is sensitive to the differing metabolic behaviours in the three trial groups. **(A)** Whole-bone averaged dynamic SUV network for the three old WT mice. **(B)** Whole-bone averaged dynamic SUV network for the three *Phospho1*^−/−^ mice. **(C)** Whole-bone averaged dynamic SUV network, including all three cohorts: *Phospho1*^−/−^ (orange), old WT (light blue), and young WT (white).

The average network created from the whole-bone 1-h dynamic SUV data for the three old WT mice, as seen in Figure 3A, shares notable properties with the young WT mice. Similar to the young WT mouse networks described in Section 3.2 and shown in Figure 2, the old WT network has dense connexions with high PCC values between long bones. It also has the humerus as a central node of the network, whereas the spine is again characterised by reduced connectivity.

While the young and old WT average SUV networks have very similar characteristics, the *Phospho1*^−/−^ network is distinctly different (Figure 3B). The humerus remains a central node to the network, but the spine has stronger correlations to other nodes and the long bones no longer have a high density of intercorrelation. In this network, the sternum is now the least similar node. However, the overall minimum PCC is notably high, meaning even the lowest correlations in the network are strongly significant.

When the average whole-bone 1-h SUV TACs from all three cohorts is put into one network, effectively combining the networks in Figures 2E, 3A, B, this produces the network in Figure 3C. Network analysis separates the data into two distinct communities within the network. One community is comprised of 8 nodes: all seven bones from the *Phospho1*^−/−^ cohort and the sternum of the young WT cohort. The other community contains 13 nodes which are the remaining WT cohort bones. This network has a 95% purity of separation between the *Phospho1*^−/−^ PET data and WT PET data. Within the WT section of the network, each of the same bones from the young and old WT mice are connected; for example, the young WT humerus is directly correlated to the old WT humerus.

## 4 Discussion

This study shows the applicability of network analysis to the study of murine bone metabolic connectivity. The many networks of young WT mice (Figure 2)—each created with different perturbations of the data—contain repetitive features that deem the analysis method robust. The networks were consistent despite shorter and longer scan times, different measurement units, and different VOI definition. Additionally, the network was robust to individual-level vs. group-level analysis. The repetitive features include the dissimilarity of the spine, the potential biological impacts of which have been discussed extensively in prior research (29). Whereas, the spine is relatively dissimilar to other bones, the humerus is a central node to each network. This connectivity means that the humerus has a very similar metabolic behaviour to many of the other bones, both axial and appendicular. The axial skeleton is comprised of bones along the central axis of the body, while appendicular bones are those comprising the upper and lower appendages. The high interconnectivity of the appendicular bones suggests they have very similar metabolic responses to the radiotracer. However, not all of the highly connected bones share the same skeletal region and function, as the sternum is also characterised by highly significant correlations to appendicular bones. It is important to note that these significances apply to the correlations between regions in the sample, but further study with a larger cohort size is necessary to make population-wide inferences about young WT mice.

There are some slight variations in the minimum PCC value across the different young WT networks. Typically, the noisier data-acquisition methods (individual mouse, cuboid) have a larger range of PCC values throughout the network. These networks thus have a lower, but still significant, minimum PCC. Despite the variations in correlation strength, the patterns across the networks remain consistent regardless of perturbations to the data. While this method of network analysis for PET quantification is robust to noise, other methods of PET quantification can be more susceptible. SUV in particular has been shown to be very affected by noise, such as causing large under and overestimations of tumour size in cancer diagnosis (6). Non-linear regression, the most complex but accurate pharmacokinetic model, is also very sensitive to noise (39). In contrast to these quantification methods, network analysis seems to be robust to noise without requiring a specific acquisition protocol.

In addition to noise, quantification of PET with SUV can be affected by scan duration. With [^18^F]FDG, uptake typically equilibrates at about 45–60 min. This means the scan start time post-injection and the duration of the scan can have significant effects on SUV results. For example, in rats, it has been shown that 45 min scans produce significantly different SUVs than 90 min scans for tumours and inflammatory lesions (40). Similar results have also been found with clinical data, where SUV and lesion detection from SUV were different for scans at ~ 1-h post-injection compared to a further 1-h delay beyond that (40, 41). Furthermore, the change in SUV over time can depend on the tissue, which is particularly relevant in total-body studies (42). Unlike SUV, pharmacokinetic analysis assesses the dynamic behaviour of radiotracer uptake, making it independent of scan length (39). Similarly, network analysis is reliant on the shape of the curve over the course of the entire scan. Network analysis utilises the data from every time point, making it resilient to the effects of scan duration beyond equilibrium.

When static SUV analysis (Figure 1D) was performed to compare the three different cohorts, no significant differences were found at the region-level. By contrast, taking the robust method of generating networks and applying it to the old WT and *Phospho1*^−/−^ cohorts, we show that the young and old WT mice have similar characteristic networks whereas the *Phospho1*^−/−^ network is distinctly different (Figures 3A, B). Additionally, when all of the data was compiled into one network, there was a 95% separation purity between nodes from WT and *Phospho1*^−/−^ data, with correlations between the same bone from each young and old WT cohort (Figure 3C). The network analysis method was sensitive to the similar metabolic behaviours across WT mice despite age. The networks were also sensitive to the differing physiological responses between the WT and *Phospho1*^−/−^ mice. It is possible that traditional SUV analysis did not show significant differences between the groups due to the limited statistical power of small cohort sizes. Network analysis is not restricted in the same way due to its reliance on dynamic data, meaning the number of time points determines the significance. However, population-wide inferences are still restricted. The reliance of network analysis on the dynamic profile of data may also account for the difference—while regions may have very similar absolute values at equilibrium, their dynamic path to equilibrium can vary greatly. Traditional SUV analysis is not sensitive to these variations. This shows that network analysis has the potential to reveal new information not otherwise found through conventional methods of PET analysis.

In a previous study of glucose uptake by the skeleton in mice, it was shown that [^18^F]FDG uptake by bone tissue decreased with age (43). Interestingly, despite this, the networks of young and old WT mice are very similar. Furthermore, the cumulative network even correlates each bone from both cohorts to one another. This is because network analysis looks at the overall curve shape rather than the specific uptake value. This allows for network analysis to assess the similarities in the active metabolic behaviour between young and old mice, rather than being dominated by the specific value.

The difference between the WT and *Phospho1*^−/−^ characteristic networks, along with the separation of the data into two components in the cumulative network, both point to PHOSPHO1 as a key regulator of metabolic processes in the bone. More generally, PHOSPHO1 has recently been shown to mediate whole-body glucose metabolism. This suggests that it would be interesting to go a step further and analyse non-bone VOIs with networks to assess the role of PHOSPHO1 and the skeleton in whole-body endocrine regulation (27, 28). This is one of many recent results displaying the important role of bone as a metabolic regulator (44, 45).

These results relate to human health particularly due to the links observed between PHOSPHO1 and disorders of altered energy metabolism in humans (21). There exists a class of compounds capable of inhibiting PHOSPHO1 in humans, which could be used to further study the phosphatase's role in both skeletal and systemic metabolism (21, 46). Studying how metabolic networks are affected by PHOSPHO1 inhibition—particularly when comparing healthy patients to those with diabetes or obesity—could affect disease understanding, diagnosis, and treatment methods. Beyond this, the effect of ageing on the WT networks could also have implications for research in humans. Mice have similar cortical bone development to humans, increasing in thickness until a certain age after which it decreases continuously (47, 48). All mice here were younger than the age when decreasing thickness is typically observed for the given strain (48). This means the findings here could be related to metabolic patterns in human cortical bone throughout the growth stage. Understanding the characteristic bone metabolic networks of young, healthy mice and humans could affect the way we approach treating low bone density and even age-related bone loss.

This study has some limitations, namely: the small cohort sizes, not having *Phospho1*^−/−^ mice age-matched to the young WT mice, and the fact that general anaesthesia was used. The small size of the cohorts decreases the significance of biological inferences. The Pearson correlation network results are still statistically significant and informative, as Pearson is dependent on the number of data points in the TACs and not the number of mice. However, the cohort sizes are too small to accurately represent a population. Additionally, having no young *Phospho1*^−/−^ mice makes it challenging to control for the potentially confounding variable of age. Previous studies have shown that the spine has reduced [^18^F]FDG uptake with age in mice, and that glucose metabolism changes with age in humans, meaning that age could affect the results of this study (43, 49). Furthermore, general anaesthesia (isoflurane), which was required to take dynamic images of mice, has been shown to affect the uptake of [^18^F]FDG and thus may have influenced the results (50). Finally, while Pearson correlation—along with covariance—has been used in many of the metabolic network analysis studies performed to date, it assumes linearity between regions (11, 13–15, 17, 18). This is not necessarily the case with [^18^F]FDG uptake. We explore the effect of using Spearman instead of Pearson in Supplementary Figure 2, which measures monotonicity without assuming linearity.

Beyond these limitations, the study also could have benefitted from the addition of human data to better contextualise the results in terms of systems biology and current clinical research. This is difficult as total-body PET in humans is still quite new and not often taken dynamically at present. It would also be beneficial to consider other radiotracers, particularly in the context of further assessing the robustness of network analysis with TBPET. An example is [^18^F]NaF which is used as a marker of calcification in the body, making it relevant for studying PHOSPHO1 and bone mineralization (51).

## 5 Conclusion

Network analysis using the method described in this paper provides a robust, statistically significant way to analyse [^18^F]FDG dynamic total-body PET data in mice, even when traditional SUV analysis fails. The method was shown to be sensitive to similar and different metabolic responses across three different mouse cohorts. Network analysis can be employed with different PET measures and dynamic or static data, allowing for easy application alongside other methods of analysis. Applying this method to larger sample sizes, more regions, different radiotracers, clinical data, and many other variations has the potential to reveal novel information about physiology and tissue-tissue interactions at a systemic level.

## Data Availability

The raw data supporting the conclusions of this article will be made available by the authors, without undue reservation.

## References

[1] Cherry SR Jones T Karp JS Qi J Moses WW Badawi RD. Total-body PET: maximizing sensitivity to create new opportunities for clinical research and patient care. J Nucl Med. (2018) 59:3–12. 10.2967/jnumed.116.184028 28935835 PMC5750522

[2] Pantel AR Viswanath V Daube-Witherspoon ME Dubroff JG Muehllehner G Parma MJ. PennPET explorer: human imaging on a whole-body imager. J Nucl Med. (2019) 61:144–51. 10.2967/jnumed.119.231845 31562224 PMC6954463

[3] Alberts I Hünermund JN Prenosil G Mingels C Bohn KP Viscione M. Clinical performance of long axial field of view PET/CT: a head-to-head intra-individual comparison of the Biograph Vision Quadra with the Biograph Vision PET/CT. Eur J Nucl Med Mol Imaging. (2021) 48:2395–404. 10.1007/s00259-021-05282-7 33797596 PMC8241747

[4] Sha L Li Y Zhang Y Tang Y Li B Chen Y. Heart-brain axis: association of congenital heart abnormality and brain diseases. Front Cardiovasc Med. (2023) 10:1071820. 10.3389/fcvm.2023.1071820 37063948 PMC10090520

[5] Bai B Bading J Conti PS. Tumor quantification in clinical positron emission tomography. Theranostics. (2013) 3:787–801. 10.7150/thno.5629 24312151 PMC3840412

[6] Boellaard R Krak N Hoekstra O Lammertsma A. Effects of noise, image resolution, and ROI definition on the accuracy of standard uptake values: a simulation study. J Nucl Med. (2004) 45:1519–27. Available online at: https://jnm.snmjournals.org/content/45/9/1519 15347719

[7] Boellaard R. Standards for PET Image Acquisition and Quantitative Data Analysis. J Nucl Med. (2009) 50:11S–20S. 10.2967/jnumed.108.057182 19380405

[8] Lammertsma AA. Forward to the Past: The Case for Quantitative PET Imaging. J Nucl Med. (2017) 58:1019–24. 10.2967/jnumed.116.188029 28522743

[9] Bullmore E Sporns O. Complex brain networks: graph theoretical analysis of structural and functional systems. Nat Rev Neurosci. (2009) 10:186–98. 10.1038/nrn2575 19190637

[10] Yakushev I Drzezga A Habeck C. Metabolic connectivity: methods and applications. Curr Opin Neurol. (2017) 30:677. 10.1097/WCO.0000000000000494 28914733

[11] Veronese M Moro L Arcolin M Dipasquale O Rizzo G Expert P. Covariance statistics and network analysis of brain PET imaging studies. Sci Rep. (2019) 9:2496. 10.1038/s41598-019-39005-8 30792460 PMC6385265

[12] Shim HK Lee HJ Kim SE Lee BI Park S Park KM. Alterations in the metabolic networks of temporal lobe epilepsy patients: a graph theoretical analysis using FDG-PET. NeuroImage. (2020) 27:102349. 10.1016/j.nicl.2020.102349 32702626 PMC7374556

[13] Sala A Perani D. Brain molecular connectivity in neurodegenerative diseases: recent advances and new perspectives using positron emission tomography. Front Neurosci. (2019) 13:617. 10.3389/fnins.2019.00617 31258466 PMC6587303

[14] Ruan J Wu Y Wang H Huang Z Liu Z Yang X. Graph theory analysis of a human body metabolic network: a systematic and organ-specific study. Med Phys. (2025) 52:2340–55. 10.1002/mp.17568 39680791

[15] Sun T Wang Z Wu Y Gu F Li X Bai Y. Identifying the individual metabolic abnormities from a systemic perspective using whole-body PET imaging. Eur J Nucl Med Mol Imaging. (2022) 49:2994–3004. 10.1007/s00259-022-05832-7 35567627 PMC9106794

[16] Yu J Spielvogel C Haberl D Jiang Z Özer Y Pusitz S. Systemic metabolic and volumetric assessment via whole-body [18F]FDG-PET/CT: pancreas size predicts cachexia in head and neck squamous cell carcinoma. Cancers. (2024) 16:3352. 10.3390/cancers16193352 39409971 PMC11475137

[17] Reed MB Ponce de León M Vraka C Rausch I Godbersen GM Popper V. Whole-body metabolic connectivity framework with functional PET. NeuroImage. (2023) 271:120030. 10.1016/j.neuroimage.2023.120030 36925087

[18] Dias AH Hansen AK Munk OL Gormsen LC. Normal values for 18F-FDG uptake in organs and tissues measured by dynamic whole body multiparametric FDG PET in 126 patients. Eur J Nucl Med Mol Imaging. (2022) 12:15. 10.1186/s13550-022-00884-0 35254514 PMC8901901

[19] Sundar LKS Hacker M Beyer T. Whole-Body PET imaging: a catalyst for whole-person research? J Nucl Med. (2023) 64:197–9. 10.2967/jnumed.122.264555 36460342 PMC9902855

[20] Guntur AR Rosen CJ. Bone as an endocrine organ. Endocrine Practice. (2012) 18:758–62. 10.4158/EP12141.RA 22784851 PMC3571654

[21] Dillon S Staines KA Millán JL Farquharson C. How to build a bone: PHOSPHO1, biomineralization, and beyond. JBMR Plus. (2019) 3:e10202. 10.1002/jbm4.10202 31372594 PMC6659447

[22] Willmer T Johnson R Louw J Pheiffer C. Blood-based DNA methylation biomarkers for type 2 diabetes: potential for clinical applications. Front Endocrinol. (2018) 9:744. 10.3389/fendo.2018.00744 30564199 PMC6288427

[23] Chambers JC Loh M Lehne B Drong A Kriebel J Motta V. Epigenome-wide association of DNA methylation markers in peripheral blood from Indian Asians and Europeans with incident type 2 diabetes: a nested case-control study. J Nucl Med. (2015) 3:526–34. 10.1016/S2213-8587(15)00127-8 26095709 PMC4724884

[24] Dayeh T Tuomi T Almgren P Perfilyev A Jansson PA de Mello VD. DNA methylation of loci within ABCG1 and PHOSPHO1 in blood DNA is associated with future type 2 diabetes risk. Epigenetics. (2016) 11:482–8. 10.1080/15592294.2016.1178418 27148772 PMC4939923

[25] Wu Y Duan H Tian X Xu C Wang W Jiang W. Genetics of obesity traits: a bivariate genome-wide association analysis. Front Genet. (2018) 9:179. 10.3389/fgene.2018.00179 29868124 PMC5964872

[26] Sayols-Baixeras S Subirana I Lluis-Ganella C Civeira F Roquer J Do A. Identification and validation of seven new loci showing differential DNA methylation related to serum lipid profile: an epigenome-wide approach. The REGICOR study. Hum Mol Genet. (2016) 25:4556–65. 10.1093/hmg/ddw285 28173150 PMC6284258

[27] Suchacki KJ Morton NM Vary C Huesa C Yadav MC Thomas BJ. PHOSPHO1 is a skeletal regulator of insulin resistance and obesity. BMC Bio. (2020) 18:149. 10.1186/s12915-020-00880-7 33092598 PMC7584094

[28] Jiang M Chavarria TE Yuan B Lodish HF Huang NJ. Phosphocholine accumulation and PHOSPHO1 depletion promote adipose tissue thermogenesis. Proc Natl Acad Sci. (2020) 117:15055–65. 10.1073/pnas.1916550117 32554489 PMC7334538

[29] Suchacki KJ Alcaide-Corral CJ Nimale S Macaskill MG Stimson RH Farquharson C. A systems-level analysis of total-body PET data reveals complex skeletal metabolism networks *in vivo*. Front Med. (2021) 8:740615. 10.3389/fmed.2021.740615 34616758 PMC8488174

[30] Yadav MC Simão AMS Narisawa S Huesa C McKee MD Farquharson C. Loss of skeletal mineralization by the simultaneous ablation of PHOSPHO1 and alkaline phosphatase function: a unified model of the mechanisms of initiation of skeletal calcification. J Bone Miner Res. (2011) 26:286–97. 10.1002/jbmr.195 20684022 PMC3179344

[31] Jacobson O Kiesewetter DO Chen X. Fluorine-18 radiochemistry, labeling strategies and synthetic routes. Bioconjug Chem. (2015) 26:1–18. 10.1021/bc500475e 25473848 PMC4306521

[32] PMOD Base Functionality, Installation Manual Version 4.4. PMOD Technologies LLC. (2022). Available online at: https://doc.pmod.com/PDF/PBAS.pdf (accessed June 2024).

[33] Suchacki KJ Tavares AAS Mattiucci D Scheller EL Papanastasiou G Gray C. Bone marrow adipose tissue is a unique adipose subtype with distinct roles in glucose homeostasis. Nat Commun. (2020) 11:3097. 10.1038/s41467-020-16878-2 32555194 PMC7303125

[34] Freeman TC Horsewell S Patir A Harling-Lee J Regan T Shih BB. Graphia: A platform for the graph-based visualisation and analysis of high dimensional data. PLoS Comput Biol. (2022) 18:1010310. 10.1371/journal.pcbi.1010310 35877685 PMC9352203

[35] Kolaczyk ED. “Ch. 7 network topology inference.• In: Kolaczyk ED, editor. Statistical Analysis of Network Data: Methods and Models. New York, NY: Springer (2009). p. 1–48.

[36] Altman DG. “Ch. 11.7 correlation - mathematics and worked examples." In: *Practical Statistics for Medical Research*. Milton, United Kingdom: CRC Press LLC (1990). p. 293–4.

[37] Altman NS. An introduction to kernel and nearest-neighbor nonparametric regression. Am Stat. (1992) 46:175–85.

[38] Girvan M Newman MEJ. Community structure in social and biological networks. Proc Natl Acad Sci. (2002) 99:7821–6. 10.1073/pnas.122653799 12060727 PMC122977

[39] Lammertsma AA Hoekstra CJ Giaccone G Hoekstra OS. How should we analyse FDG PET studies for monitoring tumour response? Eur J Nucl Med Mol Imaging. (2006) 33:16–21. 10.1007/s00259-006-0131-5 16763817

[40] Zhuang H Pourdehnad M Lambright ES Yamamoto AJ Lanuti M Li P. Dual time point 18F-FDG PET imaging for differentiating malignant from inflammatory processes. J Nucl Med. (2001) 42:1412–7. Available online at: https://jnm.snmjournals.org/content/42/9/1412.long 11535734

[41] Wu Y Fu F Meng N Wang Z Li X Bai Y. The role of dynamic, static, and delayed total-body PET imaging in the detection and differential diagnosis of oncological lesions. Cancer Imaging. (2024) 24:2. 10.1186/s40644-023-00649-5 38167538 PMC10759379

[42] Cheng G Alavi A Lim E Werner TJ Del Bello CV Akers SR. Dynamic changes of FDG uptake and clearance in normal tissues. Mol Imaging Biol. (2013) 15:345–52. 10.1007/s11307-012-0600-0 23090853

[43] Zoch ML Abou DS Clemens TL Thorek DLJ Riddle RC. In vivo radiometric analysis of glucose uptake and distribution in mouse bone. Bone Res. (2016) 4:1–8. 10.1038/boneres.2016.4 27088042 PMC4820746

[44] Lee NK Sowa H Hinoi E Ferron M Ahn JD Confavreux C. Endocrine regulation of energy metabolism by the skeleton. Cell. (2007) 130:456–69. 10.1016/j.cell.2007.05.047 17693256 PMC2013746

[45] Zhou R Guo Q Xiao Y Guo Q Huang Y Li C. Endocrine role of bone in the regulation of energy metabolism. Bone Res. (2021) 9:1–19. 10.1038/s41413-021-00142-4 34016950 PMC8137703

[46] Liu Y Wu Y Jiang M. The emerging roles of PHOSPHO1 and its regulated phospholipid homeostasis in metabolic disorders. Front Physiol. (2022) 13:935195. 10.3389/fphys.2022.935195 35957983 PMC9360546

[47] Jilka RL. The relevance of mouse models for investigating age-related bone loss in humans. J Gerontol A Biol Sci Med Sci. (2013) 68:1209–17. 10.1093/gerona/glt046 23689830 PMC3779631

[48] Halloran BP Ferguson VL Simske SJ Burghardt A Venton LL Majumdar S. Changes in Bone Structure and Mass With Advancing Age in the Male C57BL/6J Mouse*. J Bone Miner Res. (2002) 17:1044–50. 10.1359/jbmr.2002.17.6.1044 12054159

[49] Kalyani RR Egan JM. Diabetes and altered glucose metabolism with aging. Endocrinol Metab Clin North Am. (2013) 42:333–47. 10.1016/j.ecl.2013.02.010 23702405 PMC3664017

[50] Fueger BJ Czernin J Hildebrandt I Tran C Halpern BS Stout D. Impact of animal handling on the results of 18F-FDG PET studies in mice. J Nucl Med. (2006) 47:999–1006. Available online at: https://jnm.snmjournals.org/content/47/6/999.long 16741310

[51] Grant FD Fahey FH Packard AB Davis RT Alavi A Treves ST. Skeletal PET with 18F-fluoride: applying new technology to an old tracer. J Nucl Med. (2008) 49:68–78. 10.2967/jnumed.106.037200 18077529

