# Supplementary material for: Distinct bone metabolic networks identified in Phospho1−/− mice vs. wild type mice using [18F]FDG total-body PET
Source: Front Med (Lausanne). 2025 May 21;12:1597844. doi: 10.3389/fmed.2025.1597844 (PMC12133898; doi:10.3389/fmed.2025.1597844)

Supplementary Figure 1: Group SUV curves of each whole-bone region for each of the trial groups: *Phospho1*$^{-/-}$ (blue), old WT (red), and young WT (green). Data are presented as mean±SEM.

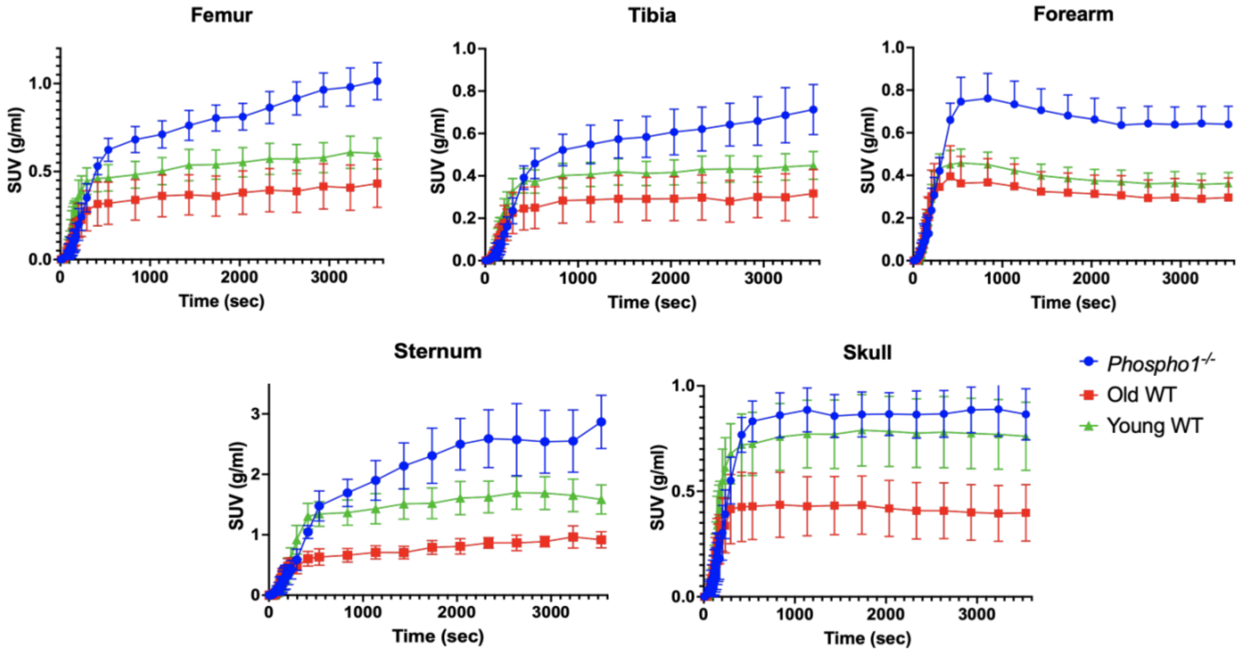

Supplementary Figure 2: Here we present networks for each of the three trial groups (calculated with 60-min SUV TACs) using Spearman instead of Pearson, which measures monotonicity and does not assume linearity. The results are effectively the same for the young WT cohort. The old WT cohort, however, is slightly different. Interestingly, we see that the Skull is now the most dissimilar node in the network, and the Spine has higher correlations. However, we do see the same increase in overall connectivity (from the young WT) as we do in the Pearson networks. We also see the same high Femur/Tibia/Humerus/Sternum correlations. In the *Phospho1*$^{-/-}$ network, we again see the overall increased correlation between all bones with a very central Humerus node. In general, it seems that most results are the same between Pearson and Spearman except for the increased Spine correlation in the old WT.

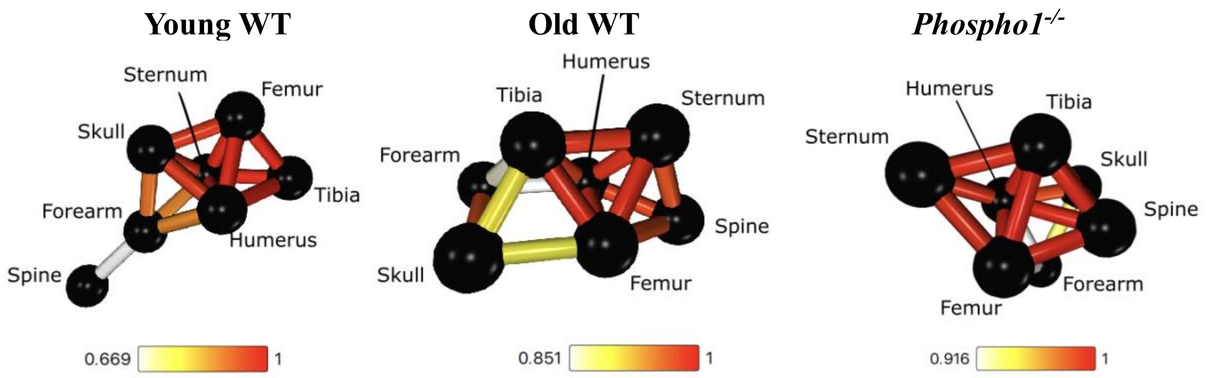

Supplement: Supplementary file 1 [file Data_Sheet_1.pdf]
